# Nematicity with a twist: Rotational symmetry breaking in a moiré superlattice

**DOI:** 10.1126/sciadv.aba8834

**Published:** 2020-08-05

**Authors:** Rafael M. Fernandes, Jörn W. F. Venderbos

**Affiliations:** 1 School of Physics and Astronomy, University of Minnesota, Minneapolis, MN 55455, USA.; 2 Department of Physics, Drexel University, Philadelphia, PA 19104, USA.; 3 Department of Materials Science and Engineering, Drexel University, Philadelphia, PA 19104, USA.

## Abstract

Motivated by recent reports of nematic order in twisted bilayer graphene (TBG), we investigate the impact of the triangular moiré superlattice degrees of freedom on nematicity. In TBG, the nematic order parameter is not Ising like, as in tetragonal crystals, but has a three-state Potts character related to the threefold rotational symmetry (*C*_3*z*_) of the moiré superlattice. We find that, even in the presence of static strain that explicitly breaks the *C*_3*z*_ symmetry, the system can still undergo a nematic-flop phase transition that spontaneously breaks in-plane twofold rotations. Moreover, elastic fluctuations, manifested as acoustic phonons, mediate a nemato-orbital coupling that ties the nematic director orientation to certain soft directions in momentum space, rendering the Potts-nematic transition mean field and first order. In contrast to the case of rigid crystals, the Fermi surface hot spots associated with these soft directions are maximally coupled to low-energy nematic fluctuations in the moiré superlattice case.

## Teaser

Twisted 2D materials realize a unique electronic nematic state, quite different from its counterpart in bulk quantum materials.

## INTRODUCTION

Recent experiments in twisted bilayer graphene (TBG) have unveiled a rich phase diagram displaying phenomena often observed in strongly correlated systems, such as superconductivity, metal-insulator transition, nematicity, ferromagnetism, and the anomalous quantum Hall effect (*1*–*7*). In contrast to correlated materials, however, in TBG, these phases can be precisely tuned with gating and twist angles, rather than doping and pressure. This offers a compelling venue to elucidate correlation-driven electronic orders (*8*–*21*), which avoids the typical complications related to bulk compounds.

In correlated materials, nematic order—a correlation-driven lowering of the point group symmetry of a crystal—is often associated with unconventional superconductivity (*22*, *23*). In TBG, scanning tunneling microscopy (STM) (*24*–*26*) and transport measurements (*27*) reported evidence that the threefold rotational symmetry of the moiré superlattice, denoted by *C*_3*z*_, is broken in different regions of the TBG phase diagram, including close to the superconducting dome. Moreover, spontaneous *C*_3*z*_ symmetry breaking has been invoked to explain the observed Landau level degeneracy at charge neutrality (*28*, *29*). While a number of theoretical proposals have explored the possibility of such an electronic nematic phase (*30*–*34*), experimentally, it remains a difficult task to distinguish spontaneous nematic order from an explicit broken symmetry caused by strain, whose presence is ubiquitous in TBG (*26*, *35*–*37*).

**Fig. 1:**
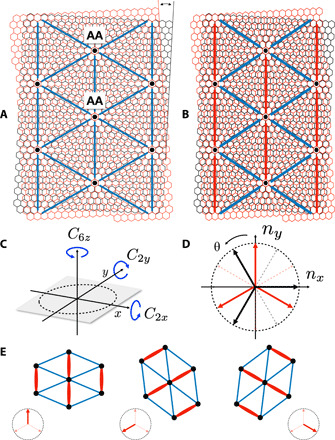
Nematic order in a moiré superlattice. (**A**) The triangular moiré superlattice of TBG (blue bonds), formed by the AA stacking regions (black dots). (**B**) In the presence of nematic order, one of the superlattice bonds becomes different (red bond), while the other two remain equivalent. (**C**) The symmetries of the moiré superlattice involve twofold rotations with respect to orthogonal in-plane axes (*C*_2*x*_ and *C*_2*y*_) and sixfold rotations with respect to the *z* axis (*C*_6*z*_). (**D**) Nematic director $\hat{n}=(\text{cos}\theta ,\text{sin}\theta )$: black (red) corresponds to γ < 0 (γ > 0) in the action in Eq. 1. Note that $\hat{n}$ and $-\hat{n}$ (dashed arrows) are identified. (**E**) Bond order pattern and lattice distortion pattern associated with each nematic director.

An important question is whether nematicity in TBG is a straightforward generalization of nematicity in bulk correlated systems or whether new effects arise from the unique properties of twisted systems. A defining property of nematic order is its strong coupling to the lattice. Because the moiré superlattice in TBG is triangular (see Fig. 1A), nematic order is not described by an Ising order parameter, as it is the case in tetragonal pnictides and cuprates (*22*), but rather by a three-state Potts model order parameter, associated with the three *C*_3*z*_-related lattice bonds (see Fig. 1B). Importantly, the moiré superlattice is an emergent lattice, whose elastic properties are fundamentally distinct than the rigid crystals of correlated materials (*38*, *39*).

Here, we show that nematicity in twisted systems can be fundamentally different than in bulk correlated systems. While static strain completely smears the nematic transition in tetragonal lattices, it allows the moiré superlattice to still undergo an Ising-like nematic-flop transition, in which in-plane twofold rotational symmetries are spontaneously broken. Finite-momentum strain fluctuations, manifested as acoustic phonons, mediate a nonanalytic nemato-orbital coupling in the moiré superlattice. The latter makes certain directions in momentum space, which are tied to the nematic director’s orientation, softer than others across the nematic transition. This is expected to render the three-state Potts-nematic transition mean field and first order and also constrains the electronic states that can exchange low-energy nematic fluctuations to a discrete set of Fermi surface hot spots. Because the moiré superlattice is not a rigid crystal (*38*, *39*), the nematic form factor is maximum at these hot spots. This contrasts with rigid lattices, where the form factor vanishes at the hot spots, effectively decoupling the electronic system from low-energy nematic fluctuations. Thus, the maximum coupling between hot spots and nematic fluctuations makes moiré superlattices promising systems to elucidate the impact of nematicity on electronic properties, such as transport and superconductivity.

## RESULTS

Electronic nematic order is described by a traceless symmetric tensor, which, in two dimensions, has two independent components Φ_1_ and Φ_2_ corresponding to the charge quadrupole moments with *d*_*x2−y2*_ and *d_xy_* symmetries, respectively. In systems with tetragonal symmetry, these two *d*-waves have distinct symmetries and must thus be treated as two independent Ising order parameters. This is markedly different in hexagonal systems, such as TBG with point group *D*_6_: The two nematic components belong to a single irreducible representation of *D*_6_ and transform as partners under its symmetries, defining a two-component order parameter **Φ** = (Φ_1_, Φ_2_). It is natural to parametrize it as **Φ** = Φ(cos2θ, sin2θ), where the angle θ can be identified with the orientation of the nematic director $\hat{n}=(\text{cos}\theta ,\text{sin}\theta )$ (see Fig. 1D); note that **Φ**(θ) = **Φ**(θ + π), as expected. Note that all of this remains true if one considers a TBG model with *D*_3_ point group symmetry instead.

Although this parametrization might suggest that **Φ** is an *XY* order parameter, the lattice symmetries of TBG introduce crystal anisotropy effects that pin the nematic director to a discrete set of high-symmetry directions. The Landau-type action *S*_nem_[**Φ**] is [see also (*30*, *40*–*42*)]

$$S_{\text{nem}}[\Phi ]=S_{0}[\Phi ]+\frac{\gamma }{6}\int_{x}(\Phi_{+}^{3}+\Phi_{-}^{3})$$ (1)

where *x* = (**r**, τ) denotes spatial coordinate **r** and imaginary time τ, and Φ_±_ ≡ Φ_1_ ± *i*Φ_2_. The first term, $S_{0}[\Phi ]=\frac{1}{2}r_{\Phi }{|\Phi |}^{2}+\frac{1}{4}u_{\Phi }{|\Phi |}^{4}$, is a standard Φ^4^ action with *U*(1) symmetry. The cubic term reflects the crystalline anisotropy of the hexagonal lattice and is expressed as $\frac{1}{3}\gamma \Phi^{3}\text{cos}6\theta$, which is minimized by θ = 2*n*π/6 for γ < 0 and θ = (2*n* + 1)π/6 for γ > 0. These solutions correspond to sets of threefold degenerate nematic directors, as shown in Fig. 1D [recall that angles differing by π (dashed arrows) must be identified], and manifested as bond orders in real space (Fig. 1E). Equation 1 is the continuum version of the three-state Potts model, where the Z_3_ symmetry is identified with the threefold rotation about the *z* axis, *C*_3*z*_. Below the nematic transition temperature *T*_nem_, the sixfold rotation symmetry *C*_6*z*_ about the *z* axis is broken down to a twofold symmetry *C*_2*z*_, while the twofold rotation symmetries *C*_2*x*_ and *C*_2*y*_ with respect to the in-plane *x* and *y* axes (or their symmetry-related equivalents) are preserved (see Fig. 1C). Despite the presence of a cubic term in (Eq. 1), the three-state Potts transition is continuous in two dimensions.

### Static strain

According to the Landau theory of phase transitions, once threefold rotation symmetry *C*_3*z*_ is broken because of condensation of the nematic order parameter **Φ**, all order parameters that also break *C*_3*z*_ symmetry and linearly couple to **Φ** will become nonzero—irrespective of their microscopic origin (be it electronic or structural). For the triangular moiré superlattice, nematic order implies a unit cell–preserving redistribution of charge between nearest-neighbor bonds, making one bond inequivalent from the remaining two. This is shown in Fig. 1E and is referred to as bond order. We emphasize that, due to the three-state Potts model nature of this order, two of the three bonds remain equivalent. Depending on the sign of the nematoelastic coupling, the distinct bond, shown in red in Fig. 1E, is either stronger or weaker compared to the other two bonds, as we discuss below.

As a result, each one of the three allowed electronic nematic directors (black or red arrows in Fig. 1D) corresponds to selecting one of the three bonds in real space. In the nematic state, the “red” bonds form unidirectional stripes along the moiré superlattice, as shown in Fig. 1B. Such a pattern was recently observed in STM experiments in TBG (*26*). Despite the appearance of stripes, this type of charge redistribution pattern is described by a zero-momentum order parameter, since there is no additional translational symmetry breaking.

To quantitatively account for the coupling between the electronic and lattice degrees of freedom, we include the latter via the strain tensor $\varepsilon_{\mathrm{ij}}\equiv \frac{1}{2}(\partial_{i}u_{j}+\partial_{j}u_{i})$ and the rotation tensor $\omega_{\mathrm{ij}}\equiv \frac{1}{2}(\partial_{i}u_{j}-\partial_{j}u_{i})$, which are defined in terms of the moiré superlattice displacement vector **u**. In the present case, the electronic nematic order parameter **Φ** may refer to any fermionic bilinear that breaks *C*_3*z*_ symmetry, which could be in the charge, valley, or spin channel. On the other hand, ε*_ij_* and ω*_ij_* refer only to the lattice geometric degrees of freedom. The elasto-nematic action is given by $S_{\text{el}-\text{nem}}[\Phi ,\hat{\varepsilon },\hat{\omega }]=S_{\text{el}}[\hat{\varepsilon },\hat{\omega }]+S\prime [\Phi ,\hat{\varepsilon }]$, where $S_{\text{el}}[\hat{\varepsilon },\hat{\omega }]$ is the elastic free energy and

$$S\prime [\Phi ,\hat{\varepsilon }]=-\lambda \int_{x}[(\varepsilon_{\mathrm{xx}}-\varepsilon_{\mathrm{yy}})\Phi_{1}+2\varepsilon_{\mathrm{xy}}\Phi_{2}]$$ (2)

describes the nematoelastic coupling with coupling constant λ.

**Fig. 2:**
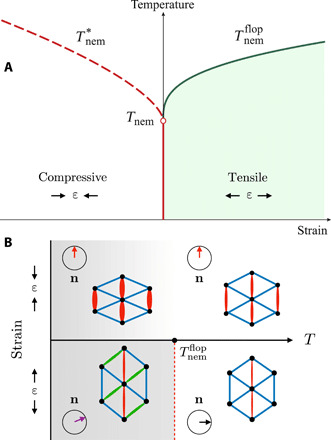
Nematic transition in the presence of static strain. (**A**) Schematic temperature versus strain phase diagram with strain applied along the *y* axis (α = π/2) and λ < 0, γ > 0. For compressive strain (ε < 0), because the director is fixed at θ_0_ = π/2, which is a minimum of the cubic term, no phase transition occurs, and only a crossover temperature $T_{\text{nem}}^{*}$ survives. For tensile strain (ε > 0), the director is at θ_0_ = 0, which is a maximum of the cubic term, for $T>T_{\text{nem}}^{\text{flop}}$, and at $\pm {\bar{\theta }}_{0}$ for $T<T_{\text{nem}}^{\text{flop}}$. Thus, $T_{\text{nem}}^{\text{flop}}$ marks an Ising-like nematic-flop transition in which the twofold rotational symmetries *C*_2*x*_ and *C*_2*y*_ are spontaneously broken. The sixfold rotation *C*_6*z*_ is explicitly broken to *C*_2*z*_ everywhere for ε ≠ 0. (**B**) Evolution of the bond order patterns as temperature is lowered in the cases of compressive and tensile strain. Only in the latter case, the three bonds become inequivalent at low temperatures, signaling the breaking of *C*_2*x*_ and *C*_2*y*_.

We consider first the effect of static strain applied in TBG. For compressive (tensile) uniaxial strain ε < 0 (ε > 0) applied parallel to an arbitrary direction $\hat{d}$, the action above becomes *S*′ = − λ∫*_x_*ε Φ cos(2α − 2θ), where $\text{cosα}=\hat{d}\cdot \hat{x}$. At high temperatures *T* ≫ *T*_nem_, where *T*_nem_ is the transition temperature of the unstrained system, we can approximate $S_{\text{nem}}\approx \frac{1}{2}\int_{x}\chi_{\text{nem}}^{-1}\Phi^{2}$. Thus, strain not only triggers a finite nematic order parameter Φ ∝ χ_nem_∣ε∣ but also pins the nematic director parallel or perpendicular to the strain direction; i.e., θ_0_ = α or θ_0_ = α + π/2, depending on whether λε > 0 or λε < 0, respectively. To understand what happens as temperature is lowered, we consider *T* ≪ *T*_nem_ and set ∣**Φ**∣ = Φ_0_ as approximately constant. Expanding around the high-temperature director, θ = θ_0_ + δθ, gives

$$S_{\text{nem}}+S\prime =\int_{x}[a_{\theta_{0}}(\delta \theta )+b_{\theta_{0}}{\left(\delta \theta \right)}^{2}]$$ (3)

with coefficients $a_{\theta_{0}}=-2\gamma \Phi_{0}^{3}\text{sin}6\theta_{0}$ and $b_{\theta_{0}}=2\Phi_{0}(|\lambda \varepsilon |-3\gamma \Phi_{0}^{2}\text{cos}6\theta_{0})$. Let us focus first on the case when strain is applied along a high-symmetry direction (α = *n*π/6). It follows that *a*_θ_0__ = 0 and that the system remains symmetric under twofold rotations with respect to in-plane axes, *C*_2*x*_ and *C*_2*y*_. These symmetries can nevertheless be broken spontaneously, which is signaled by *b*_θ_0__ < 0 in Eq. 3. This can only happen if θ_0_ coincides with the maxima, but not the minima, of the cubic term in Eq. 1—in other words, if the strain term *S*′ is minimized by a director that is maximally penalized by the cubic term of *S*_nem_. In this case, once Φ_0_ reaches the critical value ${\bar{\Phi }}_{0}=\sqrt{|\frac{\lambda \varepsilon }{3\gamma }|}$, usually at a temperature $T_{\text{nem}}^{\text{flop}}>T_{\text{nem}}$, the minimum changes from θ_0_ to $\theta_{0}\pm {\bar{\theta }}_{0}$, with ${\bar{\theta }}_{0}=\frac{1}{2}\text{arccos}(\frac{1}{2}\sqrt{1+\frac{3{\bar{\Phi }}_{0}^{2}}{\Phi_{0}^{2}}})$, resulting in an Ising-like transition that spontaneously breaks the *C*_2*x*_ and *C*_2*y*_ symmetries. Because of its resemblance to a spin-flop transition, we call the reorientation of the nematic director under an external field a nematic-flop transition. Thus, as illustrated in the schematic phase diagram of Fig. 2A, a nematic-related phase transition can still occur in a strained triangular lattice (*43*), in contrast to the case of a strained tetragonal lattice, where only a crossover exists.

From the perspective of bond order, the breaking of the twofold *C*_2*x*_/*C*_2*y*_ symmetries (in addition to *C*_3*z*_), makes all three bonds connecting nearest-neighbor sites inequivalent. Recall that, in the nematic phase, where only the *C*_3*z*_ symmetry is broken, two of the three bonds remain equivalent. Therefore, referring to the phase diagram of Fig. 2A, the sequence of bond order patterns observed as temperature is lowered would be the one illustrated in Fig. 2B. Without applied strain, the three bonds are equivalent. Application of compressive strain along one of the bonds would make that bond distinct and the other two equivalent. This is an explicit symmetry breaking induced by the strain. Upon lowering the temperature, no additional symmetry breaking occurs. However, for tensile strain, once the temperature becomes smaller than $T_{\text{nem}}^{\text{flop}}$, one of the two remaining equivalent bonds would become different. This is a spontaneous Ising-like symmetry breaking, since one among two bonds is chosen.

Since STM has been able to identify bond order patterns in the nematic phase (*26*), it provides a potential tool to probe such a nematic-flop transition. An appealing experiment, capable of distinguishing between spontaneous nematicity and strain-induced anisotropies, would consist of comparing the types of bond order that appear in the presence of tensile and compressive strain applied along one of the crystalline directions. If in both cases one sees unidirectional bond order patterns, then this would imply that the system is in the nematically disordered phase and that strain is causing the anisotropies. However, if in one case the type of bond order evolves as a function of temperature according to what is shown in the lower half of Fig. 2B, then this would be evidence for long-range nematic order.

Let us now consider the case when strain is not applied along one of the high-symmetry directions, i.e., α ≠ *n*π/6; then, *a*_θ_0__ ≠ 0 in Eq. 3. As a result, the applied strain breaks not only *C*_3*z*_ but also *C*_2*x*_/*C*_2*y*_ symmetry. Consequently, no true phase transition can happen. Nevertheless, near $T_{\text{nem}}^{\text{flop}}$, the nematic director θ will display strong changes as temperature is lowered, which is manifested as a sharp temperature dependence of the bond order pattern. If the system does not have a nematic ground state, however, then θ will change only weakly as a function of temperature.

### Fluctuating strain

**Fig. 3:**
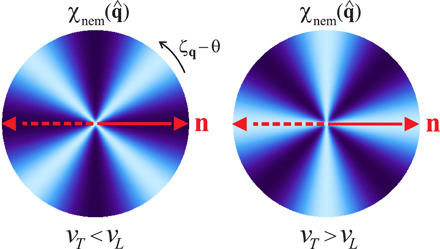
Phonon-mediated nemato-orbital coupling. Momentum directional dependence of the nematic susceptibility $\chi_{\text{nem}}(q\to 0,\hat{q})$ caused by the nemato-orbital coupling, with $\hat{q}=(\text{cos}\zeta_{q},\text{sin}\zeta_{q})$. Light blue (dark blue) denotes softer (harder) directions, corresponding to higher (lower) susceptibility. While for a rigid crystal, *v_T_* < *v_L_*, the soft direction is rotated by ±π/4 with respect to the nematic director **n** (red arrow), for TBG, *v_T_* > *v_L_*, the rotation is 0, π/2. Note that the director can point in any of the directions θ of Fig. 1B.

Apart from static strain, finite-momentum elastic fluctuations are expected to strongly affect the nematic transition (*44*–*46*). For a system with *D*_6_ symmetry, diagonalization of the harmonic elastic action $S_{\text{el}}[\hat{\varepsilon },\hat{\omega }]$ leads to two acoustic phonon modes, a transverse (*T*) and a longitudinal (*L*) one with sound velocities *v*_*L*, *T*_

$$S_{\text{el}}=\frac{1}{2}\sum_{\mu =L,T}\int_{q}{\tilde{u}}_{q,\mu }(\omega_{n}^{2}+v_{\mu }^{2}q^{2}){\tilde{u}}_{-q,\mu }$$ (4)

where *q* = (**q**, ω*_n_*), with ω*_n_* the (bosonic) Matsubara frequency. The displacement field $u=\sum_{\mu }{\tilde{u}}_{\mu }{\hat{e}}_{\mu }$ has been decomposed into its longitudinal and transverse components ${\tilde{u}}_{\mu }$ with ${\hat{e}}_{L}=(\text{cos}\zeta_{q},\text{sin}\zeta_{q})$ and ${\hat{e}}_{T}=(-\text{sin}\zeta_{q},\text{cos}\zeta_{q})$ and ζ_**q**_ = arctan (*q_y_*/*q_x_*). According to (*38*, *39*), for the dominant acoustic phonons that act on the moiré superlattice scale, **u** corresponds to the relative displacement of the two graphene sheets. These and other phonon modes have been proposed to be linked to superconductivity in TBG (*18*, *47*–*49*). Integrating out the acoustic phonons leads to an additional contribution to the nematic action, $\delta S_{\text{nem}}=-\frac{1}{2}\sum_{\mathrm{ij}}\int_{q}\;\Phi_{i,q}{\hat{\Pi }}_{\mathrm{ij}}(q)\Phi_{j,-q}$. In the static limit, ω*_n_* = 0, we find

$$\begin{array}{c} \hat{\Pi }=\frac{\lambda^{2}}{v_{T}^{2}}[\hat{I}-\eta \hat{P}],\\ \hat{P}=(\begin{array}{cc} {\text{cos}}^{2}2\zeta_{q} & \frac{1}{2}\text{sin}4\zeta_{q}\\ \frac{1}{2}\text{sin}4\zeta_{q} & {\text{sin}}^{2}2\zeta_{q} \end{array}) \end{array}$$ (5)

where $\hat{I}$ is the identity matrix and $\eta \equiv 1-v_{T}^{2}/v_{L}^{2}$. The first term of $\hat{\Pi }$ gives an overall enhancement of *T*_nem_. The second term couples the two nematic components Φ_1_ and Φ_2_ in a way that depends on the direction but not on the magnitude of **q**. Such a nonanalytic term typically appears when order parameters couple linearly to an elastic mode (*50*) and was previously studied for Ising nematic order in tetragonal lattices (*44*, *45*). Here, it is manifested as a nemato-orbital coupling

$$\begin{array}{cc} S_{\text{nem}}^{\left(\text{eff}\right)}[\Phi ] & =S_{0}[\Phi ]+\frac{\gamma }{6}\int_{x}(\Phi_{+}^{3}+\Phi_{-}^{3})\\ & +\frac{\lambda^{2}}{v_{T}^{2}}[-\int_{x}\Phi^{2}+\eta \int_{q}{\left(\Phi \cdot \hat{D}\right)}^{2}] \end{array}$$ (6)

where $\hat{D}=(\text{cos}2\zeta_{q},\text{sin}2\zeta_{q})=({\hat{q}}_{x}^{2}-{\hat{q}}_{y}^{2},2{\hat{q}}_{x}{\hat{q}}_{y})$ is the momentum space (i.e., orbital) quadrupolar form factor. Recasting the nemato-orbital coupling term as Φ^2^cos^2^(2θ − 2ζ_**q**_), we see that it makes only certain directions of momentum space to become soft; i.e., the static nematic susceptibility $\chi_{\text{nem}}(q\to 0,\hat{q})$ is largest near *T*_nem_ only along special directions $\hat{q}$ [see also (*45*)]. While the cubic term in Eq. 6 forces the director $\hat{n}$ to point along one of three directions (see Fig. 1D), the nemato-orbital coupling makes only two momentum space directions ζ_**q**_ soft, namely, the ones that make a relative angle of 0 and $\frac{\pi }{2}$ (for η < 0) or $\pm \frac{\pi }{4}$ (for η > 0) with respect to $\hat{n}$ (see Fig. 3). The reduction of the soft-direction phase space from continuous to discrete is known to effectively enhance the dimensionality of the Φ^4^ action *S*_0_[**Φ**] from *d* to *d* + 1. Thus, one expects that the nematic transition in the moiré superlattice will be the same as a three-dimensional three-state Potts model transition, which is mean field and first order.

### Electronic degrees of freedom

If the first-order character of the nematic transition discussed above is weak, then nematic fluctuations are still expected to affect the electronic degrees of freedom. For a single-band system with fermionic operator *c*_**k**_, the electronic nematic coupling is $S_{\text{elec}}=\int_{k,q}g(k)\Phi_{q}c_{k-q/2}^{†}c_{k+q/2}$, with form factor *g*(**k**) = *g*_0_ cos(2θ − 2θ_**k**_), where *g*_0_ is a constant and θ_**k**_ = arctan (*k_y_*/*k_x_*). The electronic states that exchange low-energy nematic fluctuations are at the Fermi surface and separated by the small momentum **q** of the nematic mode. Since the nematic fluctuations are the softest (albeit nondiverging) along the special directions ${\hat{q}}^{\left(0\right)}=(\text{cos}\zeta_{q}^{\left(0\right)},\text{sin}\zeta_{q}^{\left(0\right)})$ discussed above, the relevant pairs of fermions are located around the “hot spots” **k**_hs_ where the Fermi surface’s tangent is parallel to ${\hat{q}}^{\left(0\right)}$, i.e., ${\hat{q}}^{\left(0\right)}\cdot \nabla \xi_{k_{\text{hs}}}=0$. The issue is how strong these fermions are coupled to the nematic fluctuations, i.e., what the magnitude of *g*(**k**_hs_) is. For a circular Fermi surface, the hot spots are located at $\theta_{k_{\text{hs}}}=\zeta_{q}^{\left(0\right)}+\frac{\pi }{2}$, and thus $g(k_{\text{hs}})=-g_{0}\text{cos}(2\theta -2\zeta_{q}^{\left(0\right)})$. As we saw above, if η > 0, then the soft directions are $\zeta_{q}^{\left(0\right)}=\theta \pm \pi /4$, yielding *g*(**k**_hs_) = 0. Thus, in this case, the hot spots effectively decouple from the softest nematic fluctuations, similarly to what was obtained for an Ising nematic tetragonal lattice (*45*). On the other hand, if η < 0, then the soft directions are $\zeta_{q}^{\left(0\right)}=\theta ,\theta \pm \pi /2$, implying that ∣*g*(**k**_hs_)∣ = ∣*g*_0_∣; i.e., the hot spots are maximally coupled to the soft nematic fluctuations. For a generic noncircular Fermi surface respecting *D*_6_ symmetry *g*(**k**_hs_) remains maximum for η < 0 but is expected to be nonzero albeit small for η > 0.

The sign of $\eta \equiv 1-v_{T}^{2}/v_{L}^{2}$ is determined by the elastic action $S_{\text{el}}[\hat{\varepsilon },\hat{\omega }]$. For a rigid crystal, $S_{\text{el}}[\hat{\varepsilon },\hat{\omega }]=\frac{1}{2}\int_{x}[{\left(\partial_{\tau }u\right)}^{2}+C_{\text{ijkl}}\varepsilon_{\mathrm{ij}}\varepsilon_{\mathrm{kl}}]$ depends only on the strain $\hat{\varepsilon }$, since global rotations do not cost energy. In a triangular lattice, there are only two independent elastic constants, *C*_11_ ≡ *C_xxxx_* and *C*_12_ ≡ *C_xxyy_*, yielding $v_{L}^{2}=C_{11}$ and $v_{T}^{2}=(C_{11}-C_{12})/2$. Lattice stability requires *C*_11_ > ∣*C*_12_∣, which makes η > 0, implying that *g*(**k**_hs_) is small. However, the moiré superlattice is not a rigid crystalline structure for small twist angles, as lattice relaxation leads to sharp domain walls separating the regions with AB and BA stacking. Because of this, arbitrary rotations of the moiré superlattice cost energy, and the elastic free energy acquires an extra term, $\delta S_{\text{el}}[\hat{\omega }]=\frac{1}{2}\int_{x}K\;\omega_{\mathrm{xy}}^{2}$ (*39*). This term contributes only to the transverse velocity and when *K* > 2(*C*_11_ + *C*_12_), *v_T_* becomes larger than *v_L_* (i.e., η < 0), implying that ∣*g*(**k**_hs_)∣ is maximum. Recent calculations of the acoustic phonon spectrum of TBG found that this condition is satisfied for small twist angles (*38*, *39*), making TBG a rather unique system in which the Fermi surface hot spots are maximally coupled to the nematic fluctuations.

**Fig. 4:**
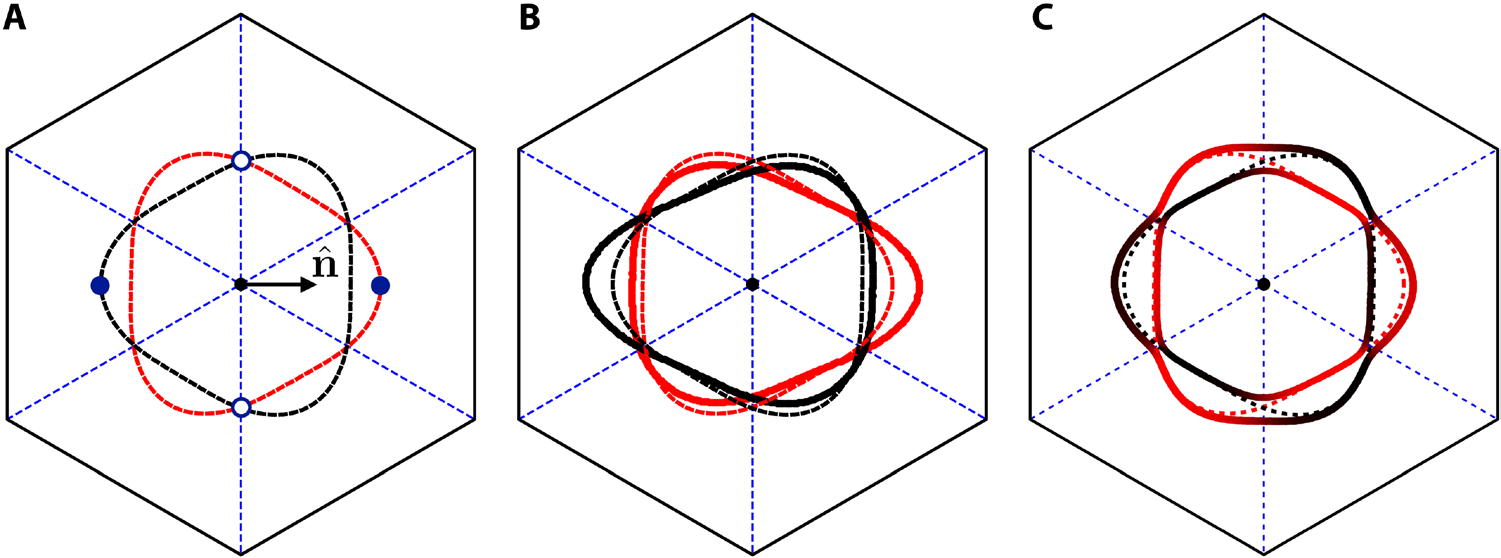
Fermi surface distortion and nematic hot spots. (**A**) Fermi surface of the six-band model in (*51*); red and black correspond to the two valleys. The two pairs of hot spots are marked by open and full symbols. (**B**) Distortion of the Fermi surface in the presence of intravalley Potts-nematic order, with nematic director **n** along the *x* axis. (**C**) Same as (B), but for intervalley nematic order. In (B) and (C), the undistorted Fermi surface is shown by the dashed lines.

To apply these results specifically to TBG, we use the six-band model in (*51*), generalizing it to the case where different types of nematic order are present (for details, see Materials and Methods). As shown in Fig. 4A, there are two Fermi surfaces associated with the two valley degrees of freedom and thus related by a *C*_2*z*_ rotation. Because the two pairs of hot spots for a given nematic director θ are related by π/2 rotations, they correspond to different valley symmetries. Setting θ = 0 for concreteness, we find that the pair of hot spots located at θ_**k**_hs__ = 0, π is associated with intravalley nematicity (Fig. 4B), whereas the pair located at θ_**k**_hs__ = ± π/2 is associated with intervalley nematicity (Fig. 4C).

## DISCUSSION

The manifestations and consequences of electronic nematicity in TBG that we have analyzed and discussed do not depend on the microscopic nature of nematic order; i.e., they do not depend on whether nematicity originates from charge, valley, or spin degrees of freedom. Nevertheless, it is important to discuss the possible microscopic mechanisms for Potts-nematic order in TBG. In weak-coupling approaches, a Pomeranchuk instability breaking the *C*_3*z*_ rotational symmetry of the Fermi surface can be favored by van Hove singularities (*52*, *53*). In strong-coupling approaches, where charge degrees of freedom are quenched, a widely used effective Hamiltonian is described in terms of an SU(4) “super spin” associated with spin and orbital variables (*21*, *30*, *54*–*58*). These orbital variables are associated with eigenstates of the *C*_3*z*_ symmetry operation and, in certain formulations, are nearly fully valley polarized (*21*). In this SU(4) formulation, nematicity is then described by an ordering of the orbital variables, i.e., ordering in the SU(2) orbital sector, which breaks spatial rotational symmetry. Whether the ground state of the effective SU(4) Hamiltonian is a nematic phase is an interesting open question. A third possible mechanism is a nematic phase that is a vestigial order of a primary electronic ordered state that breaks *C*_3*z*_ and some additional symmetry (*23*), such as *p* + p-wave/*d* + d-wave superconductivity (*30*, *34*, *40*) or charge/spin stripe density waves (*41*).

Thus, while there are several other possible ground states in TBG, a number of them are fully compatible with electronic nematic order, as they break *C*_3*z*_ symmetry (among other symmetries). The study of the detailed microscopic mechanisms responsible for the various forms of correlated behavior in TBG is currently a very active and rapidly progressing area of research. Although a detailed microscopic description is not the focus of this work, which instead focuses on the manifestations and consequences of nematic order, motivated by experimental evidence, we can nonetheless provide a rough estimate of the nematic transition temperature. We start by noting that the energy scales of the narrow band bandwidth and of the screened Coulomb repulsion are both of the order of 10 meV. Therefore, since weak-coupling calculations often find instabilities at temperatures of a few percent of the bandwidth, whereas strong-coupling calculations usually give instabilities at temperatures of a few percent of the Coulomb repulsion, a reasonable range for the nematic transition temperature would be between 1 and 10 K.

To conclude, we showed that the Potts-like character of the nematic order parameter in triangular moiré superlattices leads to unique nematic behaviors seen neither in tetragonal systems nor in rigid triangular crystals. Notably, a nematic-flop phase transition that spontaneously breaks the in-plane twofold rotational symmetries can still take place even when *C*_3*z*_ symmetry-breaking strain is applied. This can only happen, however, for one sign of strain (i.e., either compressive or tensile) and if the ground state of the unstrained system is nematic. Thus, because the breaking of the twofold in-plane rotational symmetries is manifested as a peculiar type of bond order in the triangular lattice, this makes it possible to unambiguously detect long-range nematic order in TBG even in the presence of strain.

Moreover, we showed that the emergence of a nemato-orbital coupling mediated by acoustic phonons affects not only the character of the Potts-nematic transition, which becomes mean field and first order, but also the impact of the low-energy nematic fluctuations on the electronic properties, which is maximized because of the nonrigid nature of the moiré superlattice. Such a maximal coupling between nematic fluctuations and electrons, which is, in turn, mediated by phonons, could, in principle, lead to unusual temperature dependencies of thermodynamic and transport quantities in an intermediate temperature regime, provided that the resulting first-order character of the Potts-nematic transition is weak. In this context, it is interesting to note the recent observation of linear-in-T resistivity in TBG, whose origin remains under debate (*59*, *60*). It would be desirable to investigate whether this phonon-assisted electronic nematic coupling can promote nontrivial properties of response functions. Similarly, it will be important to assess the importance of the coupling between phonons and nematic fluctuations in phonon-based pairing mechanisms that have been proposed to explain superconductivity in TBG (*18*, *47*–*49*).

## MATERIALS AND METHODS

The results discussed in the section “Electronic degrees of freedom” of the main text for the coupling between nematic fluctuations and the low-energy electronic states are quite general, as they rely on the fact that the system has sixfold rotational symmetry. To make the connection with TBG more transparent, in the main text, we also considered a specific six-band model for TBG in the presence of nematic order. Here, we provide the details of implementing nematic order in this tight-binding model, introduced in (*51*). We will generally follow the notation in (*51*), with a few exceptions.

### Six-band tight-binding model

The six-band tight-binding model in (*51*) is defined by (*p_z_*, *p*_+_, *p*_−_) orbitals on the sites of a triangular lattice and *s* orbitals on the sites of a kagome lattice. The purpose of the six-band model is to reproduce the low-energy flat bands of TBG in such a way that all symmetries manifestly present in the continuum description are respected (and are implemented locally).

Note that the six-band model describes the two low-energy flat bands originating from a single valley of the two graphene sheets forming the bilayer system. This implies that a model that includes the full set of flat low-energy bands (apart from spin) must have 12 bands: two copies of the 6-band model related by the symmetries that exchange valleys. Here, we sketch how such a model is constructed and show how it can be supplemented with the appropriate symmetry-breaking terms to account for nematic order.

We begin by recalling the definition of the six-band model. The orbital degrees of freedom can be represented by a fermion operator $\psi_{k}^{†}$ given by

$$\psi_{k}^{†}=(p_{kz}^{†},p_{k+}^{†},p_{k-}^{†},a_{k}^{†},b_{k}^{†},c_{k}^{†})$$ (7)

Note that this notation is a slight departure from the one in (*51*). In terms of these degrees of freedom, the (six-band) Hamiltonian for a single valley is given by

$$H_{k}=(\begin{array}{ccc} H_{p_{z}}+\mu_{p_{z}} & C_{p_{\pm }p_{z}}^{†} & 0\\ C_{p_{\pm }p_{z}} & H_{p_{\pm }}+\mu_{p_{\pm }} & C_{\kappa p_{\pm }}^{†}\\ 0 & C_{\kappa p_{\pm }} & H_{\kappa }+\mu_{\kappa } \end{array})$$ (8)

Here, *H_p_z__* and *H*_*p*_±__ are the subblock Hamiltonians in the *p_z_* and *p*_±_ subspaces, and *H*_κ_ describes the coupling between the kagome lattice sites. The subblocks *C_XY_* describe the couplings between the *X* and *Y* sectors (where *X*, *Y* = *p_z_*, *p*_±_, κ). The form of all these subblocks is given in (*51*), including the parameter set that we use here [see table VI in (*51*).

To promote the six-band model to a full twelve-band model, we take two copies and introduce a valley degree of freedom as

$$\Psi_{k}^{†}=(\psi_{k+}^{†},\psi_{k-}^{†})$$ (9)

where ± labels the *K* and *K*′ valleys. The full Hamiltonian $H_{k}$ is then given by

$$H_{k}=(\begin{array}{cc} H_{k} & \\ & UH_{-k}U^{†} \end{array})$$ (10)

where *H*_**k**_ is the Hamiltonian of Eq. 8 and *U* ≡ *U*_*C*_2*z*__ is the matrix representation of the twofold rotation *C*_2*z*_. Note that (*51*) uses a gauge for which *H*_**k** + **G**_ = *H*_**k**_ holds, where **G** is a reciprocal lattice vector. Equation 10 implicitly assumes a tight-binding gauge, in which case matrix representations of symmetries, in particular *U*, are momentum independent.

The Hamiltonian of Eq. 10 with the parameters specified in (*51*) defines a tight-binding model for TBG that respects all symmetries, including a *U_v_*(1) valley conservation symmetry. Various symmetry-breaking terms can be considered, and here, we are specifically interested in terms that break *C*_3*z*_ symmetry but preserve *C*_2*z*_ symmetry, as required by quadrupolar nematic order.

Note that a coupling of the valleys of the form $\delta H=\Delta \sum_{k}\psi_{k+}^{†}\psi_{k-}+H.c.$ breaks the *U_v_*(1) valley but respects all lattice symmetries as well as time-reversal symmetry. Added to the Hamiltonian of Eq. 10 it enters as an off-diagonal block.

### Rotational symmetry breaking

Consider next the rotational symmetry-breaking terms. Since the model is built from multiple degrees of freedom, there are a number of different ways in which rotation symmetry breaking can be implemented. We first focus on the triangular lattice sector of the model. Within this sector, there are two possibilities: Nematic order can occur as a result of hopping anisotropy or because of a lifting of the orbital degeneracy. To model the first possibility, we introduce the two d-wave form factors

$$\begin{array}{c} d_{k1}=\phi_{01}+\text{Re}\;\omega^{*}\;\phi_{\bar{1}\bar{1}}+\text{Re}\;\omega \;\phi_{10},+\text{c.c.}\\ d_{k2}=\text{Im}\;\omega^{*}\;\phi_{\bar{1}\bar{1}}+\text{Im}\;\omega \;\phi_{10}+\text{c.c.} \end{array}$$ (11)

where the phases ϕ*_lm_* are defined as ϕ*_lm_* = *e*^−*i***k** · (*l***a**_1_ + *m***a**_2_)^ [see (*51*)]. Here, we use the notation $\bar{l}\equiv -l$ and ω = exp (2π*i*/3). These form factors have precisely the same symmetry as (Φ_1_, Φ_2_) introduced in the main text.

The triangular lattice d-wave form factors can then be used to introduce a symmetry-breaking perturbation in the triangular lattice (*p*-orbital) sector. For instance, we can add a perturbation δ*H_p_z__* to the Hamiltonian *H_p_z__* of the *p_z_* orbital appearing in Eq. 8 given by

$$\delta H_{p_{z}}=\Phi_{1}d_{k1}+\Phi_{2}d_{k2}$$ (12)

where Φ_1,2_ are the nematic order parameters as defined in the main text. This term gives the Fermi surface distortion of Fig. 4B of the main text. Clearly, the same perturbation (but proportional to the appropriate identity matrix) can be added to *H*_*p*_±__, which describes the *p*_±_ orbitals.

In the *p*_±_-orbital sector, the nematic order parameter couples to another symmetry-breaking perturbation, which is independent of momentum. Making the two orbitals inequivalent lifts their degeneracy and necessarily breaks threefold rotation symmetry. In particular, the perturbation δ*H*_*p*_±__, which achieves this, is given by

$$\delta H_{p_{\pm }}=(\begin{array}{cc} 0 & \Phi_{1}-i\Phi_{2}\\ \Phi_{1}+i\Phi_{2} & 0 \end{array})$$ (13)

Note that the diagonal terms are zero, since time-reversal symmetry must be preserved. (An overall energy can be absorbed in μ_*p*_±__.) The Fermi surface distortion due to Eq. 13 is qualitatively similar to a distortion originating from d-wave form factors in the kinetic terms (Fig. 4B of the main text).

Consider next the kagome lattice sector of the model. The kagome sector does not have an orbital degree of freedom, but it does have multiple sites in the unit cell. The simplest coupling to the nematic order parameter is given by a charge ordering perturbation within the unit cell, which breaks threefold rotations but preserves the twofold rotation *C*_2*z*_. Specifically, the perturbation δ*H*_κ_ to the kagome lattice Hamiltonian *H*_κ_ is given by

$$\delta H_{\kappa }=(\Phi_{1}-i\Phi_{2})(\begin{array}{ccc} 1 & & \\ & \omega & \\ & & \omega^{*} \end{array})+H.c.$$ (14)

The rotational symmetry-breaking perturbations introduced so far are all intravalley perturbations; they should be considered as perturbations to Eq. 8, with the full Hamiltonian given by the prescription of Eq. 10. One may, however, also consider intervalley nematic coupling terms, which enter the off-diagonal blocks in Eq. 10. More precisely, the Hamiltonian of Eq. 10 is modified according to

$$H_{k}\to (\begin{array}{cc} H_{k} & \\ & UH_{-k}U^{†} \end{array})+\delta H_{\Phi }$$ (15)

where δH_Φ_ collects all terms that describe nematic distortions and takes the form

$$\delta H_{\Phi }=(\begin{array}{cc} & \Delta_{\Phi }\\ \Delta_{\Phi }^{†} & \end{array})$$ (16)

The form of Δ_Φ_ depends on the choice of nematic coupling; as in the case of intravalley nematic coupling, in principle, many possibilities of intervalley nematic coupling exist. One simple type of nematic coupling is given by

$$\Delta_{\Phi }=\delta H_{p_{z}}⊕\delta H_{p_{\pm }}$$ (17)

where δ*H_p_z__* and δ*H*_*p*_±__ are given by Eqs. 12 and 13.

We have based our microscopic discussion of rotation symmetry breaking on the model introduced in (*51*). This was motivated by the natural implementation of all relevant symmetries in this model. It is worth stressing that an analysis of nematic order in TBG similar to the one presented here can also be obtained from different microscopic (tight-binding) models proposed for TBG (*11*–*14*, *21*).

## References

[1] Cao Y., Fatemi V., Fang S., Watanabe K., Taniguchi T., Kaxiras E., Jarillo-Herrero P., Unconventional superconductivity in magic-angle graphene superlattices. Nature 556, 43–50 (2018). 29512651 10.1038/nature26160

[2] Cao Y., Fatemi V., Demir A., Fang S., Tomarken S. L., Luo J. Y., Sanchez-Yamagishi J. D., Watanabe K., Taniguchi T., Kaxiras E., Ashoori R. C., Jarillo-Herrero P., Correlated insulator behaviour at half-filling in magic-angle graphene superlattices. Nature 556, 80–84 (2018). 29512654 10.1038/nature26154

[3] Yankowitz M., Chen S., Polshyn H., Zhang Y., Watanabe K., Taniguchi T., Graf D., Young A. F., Dean C. R., Tuning superconductivity in twisted bilayer graphene. Science 363, 1059–1064 (2019). 30679385 10.1126/science.aav1910

[4] Lu X., Stepanov P., Yang W., Xie M., Aamir M. A., Das I., Urgell C., Watanabe K., Taniguchi T., Zhang G., Bachtold A., MacDonald A. H., Efetov D. K., Superconductors, orbital magnets and correlated states in magic-angle bilayer graphene. Nature 574, 653–657 (2019). 31666722 10.1038/s41586-019-1695-0

[5] Sharpe A. L., Fox E. J., Barnard A. W., Finney J., Watanabe K., Taniguchi T., Kastner M. A., Goldhaber-Gordon D., Emergent ferromagnetism near three-quarters filling in twisted bilayer graphene. Science 365, 605–608 (2019). 31346139 10.1126/science.aaw3780

[6] Serlin M., Tschirhart C. L., Polshyn H., Zhang Y., Zhu J., Watanabe K., Taniguchi T., Balents L., Young A. F., Intrinsic quantized anomalous Hall effect in a moiré heterostructure. Science 367, 900–903 (2020). 31857492 10.1126/science.aay5533

[7] Xie Y., Lian B., Jäck B., Liu X., Chiu C.-L., Watanabe K., Taniguchi T., Bernevig B. A., Yazdani A., Spectroscopic signatures of many-body correlations in magic-angle twisted bilayer graphene. Nature 572, 101–105 (2019). 31367031 10.1038/s41586-019-1422-x

[8] dos Santos J. M. B. L., Peres N. M. R., Neto A. H. C., Graphene Bilayer with a Twist: Electronic Structure. Phys. Rev. Lett. 99, 256802 (2007). 18233543 10.1103/PhysRevLett.99.256802

[9] Bistritzer R., MacDonald A. H., Moiré bands in twisted double-layer graphene. PNAS 108, 12233–12237 (2011). 21730173 10.1073/pnas.1108174108 PMC3145708

[10] Mele E. J., Band symmetries and singularities in twisted multilayer graphene. Phys. Rev. B 84, 235439 (2011).

[11] Yuan N. F. Q., Fu L., Model for the metal-insulator transition in graphene superlattices and beyond. Phys. Rev. B 98, 045103 (2018).

[12] Po H. C., Zou L., Vishwanath A., Senthil T., Origin of mott insulating behavior and superconductivity in twisted bilayer graphene. Phys. Rev. X 8, 031089 (2018).

[13] Koshino M., Yuan N. F. Q., Koretsune T., Ochi M., Kuroki K., Fu L., Maximally localized wannier orbitals and the extended hubbard model for twisted bilayer graphene. Phys. Rev. X 8, 031087 (2018).

[14] Zou L., Po H. C., Vishwanath A., Senthil T., Band structure of twisted bilayer graphene: Emergent symmetries, commensurate approximants, and Wannier obstructions. Phys. Rev. B 98, 085435 (2018).

[15] Kang J., Vafek O., Symmetry, maximally localized wannier states, and a low-energy model for twisted bilayer graphene narrow bands. Phys. Rev. X 8, 031088 (2018).

[16] Rademaker L., Mellado P., Charge-transfer insulation in twisted bilayer graphene. Phys. Rev. B 98, 235158 (2018).

[17] Zhang L., Lowest-energy moiré band formed by Dirac zero modes in twisted bilayer graphene. Sci. Bull. 64, 495–498 (2019). 10.1016/j.scib.2019.03.010 36659736

[18] Lian B., Wang Z., Bernevig B. A., Twisted bilayer graphene: A phonon-driven superconductor. Phys. Rev. Lett. 122, 257002 (2019). 31347876 10.1103/PhysRevLett.122.257002

[19] Wu F., Das Sarma S., Identification of superconducting pairing symmetry in twisted bilayer graphene using in-plane magnetic field and strain. Phys. Rev. B 99, 220507 (2019).

[20] Lin Y.-P., Nandkishore R. M., Chiral twist on the high-*T_c_* phase diagram in moiré heterostructures. Phys. Rev. B 100, 085136 (2019).

[21] Kang J., Vafek O., Strong coupling phases of partially filled twisted bilayer graphene narrow bands. Phys. Rev. Lett. 122, 246401 (2019). 31322361 10.1103/PhysRevLett.122.246401

[22] Fradkin E., Kivelson S. A., Lawler M. J., Eisenstein J. P., Mackenzie A. P., Nematic fermi fluids in condensed matter physics. Annu. Rev. Condens. Matter Phys. 1, 153–178 (2010).

[23] Fernandes R. M., Orth P. P., Schmalian J., Intertwined vestigial order in quantum materials: Nematicity and beyond. Annu. Rev. Condens. Matter Phys. 10, 133–154 (2019).

[24] Choi Y., Kemmer J., Peng Y., Thomson A., Arora H., Polski R., Zhang Y., Ren H., Alicea J., Refael G., von Oppen F., Watanabe K., Taniguchi T., Nadj-Perge S., Electronic correlations in twisted bilayer graphene near the magic angle. Nat. Phys. 15, 1174–1180 (2019).

[25] Kerelsky A., McGilly L. J., Kennes D. M., Xian L., Yankowitz M., Chen S., Watanabe K., Taniguchi T., Hone J., Dean C., Rubio A., Pasupathy A. N., Maximized electron interactions at the magic angle in twisted bilayer graphene. Nature 572, 95–100 (2019). 31367030 10.1038/s41586-019-1431-9

[26] Jiang Y., Lai X., Watanabe K., Taniguchi T., Haule K., Mao J., Andrei E. Y., Charge order and broken rotational symmetry in magic-angle twisted bilayer graphene. Nature 573, 91–95 (2019). 31365921 10.1038/s41586-019-1460-4

[27] Y. Cao, D. Rodan-Legrain, J. M. Park, F. N. Yuan, K. Watanabe, T. Taniguchi, R. M. Fernandes, L. Fu, P. Jarillo-Herrero, Nematicity and Competing Orders in Superconducting Magic-Angle Graphene. arXiv: 2004.04148 (2020). 10.1126/science.abc2836 33859029

[28] Zhang Y.-H., Po H. C., Senthil T., Landau level degeneracy in twisted bilayer graphene: Role of symmetry breaking. Phys. Rev. B 100, 125104 (2019).

[29] S. Liu, E. Khalaf, J. Y. Lee, A. Vishwanath, Nematic topological semimetal and insulator in magic angle bilayer graphene at charge neutrality. arXiv: 1905.07409 (2019).

[30] Venderbos J. W. F., Fernandes R. M., Correlations and electronic order in a two-orbital honeycomb lattice model for twisted bilayer graphene. Phys. Rev. B 98, 245103 (2018).

[31] Dodaro J. F., Kivelson S. A., Schattner Y., Sun X. Q., Wang C., Phases of a phenomenological model of twisted bilayer graphene. Phys. Rev. B 98, 075154 (2018).

[32] Isobe H., Yuan N. F. Q., Fu L., Unconventional superconductivity and density waves in twisted bilayer graphene. Phys. Rev. X 8, 041041 (2018).

[33] Kozii V., Isobe H., Venderbos J. W. F., Fu L., Nematic superconductivity stabilized by density wave fluctuations: Possible application to twisted bilayer graphene. Phys. Rev. B 99, 144507 (2019).

[34] Chichinadze D. V., Classen L., Chubukov A. V., Nematic superconductivity in twisted bilayer graphene. Phys. Rev. B 101, 224513 (2020)

[35] Uri A., Grover S., Cao Y., Crosse J. A., Bagani K., Rodan-Legrain D., Myasoedov Y., Watanabe K., Taniguchi T., Moon P., Koshino M., Jarillo-Herrero P., Zeldov E., Mapping the twist angle and unconventional Landau levels in magic angle graphene. Nature 581, 47 (2020) 32376964 10.1038/s41586-020-2255-3

[36] Cea T., Walet N. R., Guinea F., Electronic band structure and pinning of Fermi energy to Van Hove singularities in twisted bilayer graphene: A self-consistent approach. Phys. Rev. B 100, 205113 (2019).

[37] Wilson J. H., Fu Y., Das Sarma S., Pixley J. H., Disorder in Twisted Bilayer Graphene. Phys. Rev. Research 2, 023325 (2020)

[38] Koshino M., Son Y.-W., Moiré phonons in twisted bilayer graphene. Phys. Rev. B 100, 075416 (2019).

[39] Ochoa H., Moiré-pattern fluctuations and electron-phason coupling in twisted bilayer graphene. Phys. Rev. B 100, 155426 (2019).

[40] Hecker M., Schmalian J., Vestigial nematic order and superconductivity in the doped topological insulator Cu _x_ Bi_2_Se_3_. npj Quantum Mater. 3, 26 (2018).

[41] A. Little, C. Lee, C. John, S. Doyle, E. Maniv, N. L. Nair, W. Chen, D. Rees, J. W. Venderbos, R. Fernandes, J. G. Analytis, J. Orenstein, Observation of three-state nematicity in the triangular lattice antiferromagnet Fe_1/3_ NbS_2_ *Nat. Mater.* (2020); 10.1038/s41563-020-0681-0. 32424369

[42] S. Jin, W. Zhang, X. Guo, X. Chen, X. Zhou, X. Li, Dynamical Emergence of a Potts-Nematic Superfluid in a Hexagonal *sp*^2^ Optical Lattice. arXiv: 1910.11880 (2019).

[43] Blankschtein D., Aharony A., Effects of symmetry-breaking perturbations on the three-state Potts model. J. Phys. C Solid State Phys. 13, 4635–4648 (1980).

[44] Karahasanovic U., Schmalian J., Elastic coupling and spin-driven nematicity in iron-based superconductors. Phys. Rev. B 93, 064520 (2016).

[45] Paul I., Garst M., Lattice effects on nematic quantum criticality in metals. Phys. Rev. Lett. 118, 227601 (2017). 28621984 10.1103/PhysRevLett.118.227601

[46] de Carvalho V. S., Fernandes R. M., Resistivity near a nematic quantum critical point: Impact of acoustic phonons. Phys. Rev. B 100, 115103 (2019).

[47] Wu F., MacDonald A. H., Martin I., Theory of phonon-mediated superconductivity in twisted bilayer graphene. Phys. Rev. Lett. 121, 257001 (2018). 30608789 10.1103/PhysRevLett.121.257001

[48] Angeli M., Tosatti E., Fabrizio M., Valley jahn-teller effect in twisted bilayer graphene. Phys. Rev. X 9, 041010 (2019).

[49] Wu F., Hwang E., Das Sarma S., Phonon-induced giant linear-in-*T* resistivity in magic angle twisted bilayer graphene: Ordinary strangeness and exotic superconductivity. Phys. Rev. B 99, 165112 (2019).

[50] Cowley R. A., Acoustic phonon instabilities and structural phase transitions. Phys. Rev. B 13, 4877–4885 (1976).

[51] Po H. C., Zou L., Senthil T., Vishwanath A., Faithful tight-binding models and fragile topology of magic-angle bilayer graphene. Phys. Rev. B 99, 195455 (2019).

[52] Valenzuela B., Vozmediano M. A. H., Pomeranchuk instability in doped graphene. New J. Phys. 10, 113009 (2008).

[53] Kiesel M. L., Platt C., Thomale R., Unconventional fermi surface instabilities in the kagome hubbard model. Phys. Rev. Lett. 110, 126405 (2013). 25166827 10.1103/PhysRevLett.110.126405

[54] Xu C., Balents L., Topological superconductivity in twisted multilayer graphene. Phys. Rev. Lett. 121, 087001 (2018). 30192621 10.1103/PhysRevLett.121.087001

[55] Seo K., Kotov V. N., Uchoa B., Ferromagnetic mott state in twisted graphene bilayers at the magic angle. Phys. Rev. Lett. 122, 246402 (2019). 31322360 10.1103/PhysRevLett.122.246402

[56] Classen L., Honerkamp C., Scherer M. M., Competing phases of interacting electrons on triangular lattices in moiré heterostructures. Phys. Rev. B 99, 195120 (2019).

[57] Kiese D., Buessen F. L., Hickey C., Trebst S., Scherer M. M., Emergence and stability of spin-valley entangled quantum liquids in moiré heterostructures. Phys. Rev. Res. 2, 013370 (2020).

[58] Natori W. M. H., Nutakki R., Pereira R. G., Andrade E. C., SU(4) Heisenberg model on the honeycomb lattice with exchange-frustrated perturbations: Implications for twistronics and Mott insulators. Phys. Rev. B 100, 205131 (2019).

[59] Polshyn H., Yankowitz M., Chen S., Zhang Y., Watanabe K., Taniguchi T., Dean C. R., Young A. F., Large linear-in-temperature resistivity in twisted bilayer graphene. Nat. Phys. 15, 1011–1016 (2019). 10.1126/science.aav1910 30679385

[60] Cao Y., Chowdhury D., Rodan-Legrain D., Rubies-Bigordà O., Watanabe K., Taniguchi T., Senthil T., Jarillo-Herrero P., Strange metal in magic-angle graphene with near planckian dissipation. Phys. Rev. Lett. 124, 076801 (2020). 32142336 10.1103/PhysRevLett.124.076801

